# Current Applications of Deep Learning and Radiomics on CT and CBCT for Maxillofacial Diseases

**DOI:** 10.3390/diagnostics13010110

**Published:** 2022-12-29

**Authors:** Kuo Feng Hung, Qi Yong H. Ai, Lun M. Wong, Andy Wai Kan Yeung, Dion Tik Shun Li, Yiu Yan Leung

**Affiliations:** 1 Oral and Maxillofacial Surgery, Faculty of Dentistry, The University of Hong Kong, Hong Kong SAR, China; 2 Health Technology and Informatics, The Hong Kong Polytechnic University, Hong Kong SAR, China; 3 Imaging and Interventional Radiology, Faculty of Medicine, The Chinese University of Hong Kong, Hong Kong SAR, China; 4 Oral and Maxillofacial Radiology, Applied Oral Sciences and Community Dental Care, Faculty of Dentistry, The University of Hong Kong, Hong Kong SAR, China

**Keywords:** artificial intelligence, deep learning, radiomics, computed tomography, cone-beam computed tomography, maxillofacial diseases

## Abstract

The increasing use of computed tomography (CT) and cone beam computed tomography (CBCT) in oral and maxillofacial imaging has driven the development of deep learning and radiomics applications to assist clinicians in early diagnosis, accurate prognosis prediction, and efficient treatment planning of maxillofacial diseases. This narrative review aimed to provide an up-to-date overview of the current applications of deep learning and radiomics on CT and CBCT for the diagnosis and management of maxillofacial diseases. Based on current evidence, a wide range of deep learning models on CT/CBCT images have been developed for automatic diagnosis, segmentation, and classification of jaw cysts and tumors, cervical lymph node metastasis, salivary gland diseases, temporomandibular (TMJ) disorders, maxillary sinus pathologies, mandibular fractures, and dentomaxillofacial deformities, while CT-/CBCT-derived radiomics applications mainly focused on occult lymph node metastasis in patients with oral cancer, malignant salivary gland tumors, and TMJ osteoarthritis. Most of these models showed high performance, and some of them even outperformed human experts. The models with performance on par with human experts have the potential to serve as clinically practicable tools to achieve the earliest possible diagnosis and treatment, leading to a more precise and personalized approach for the management of maxillofacial diseases. Challenges and issues, including the lack of the generalizability and explainability of deep learning models and the uncertainty in the reproducibility and stability of radiomic features, should be overcome to gain the trust of patients, providers, and healthcare organizers for daily clinical use of these models.

## 1. Introduction

**Figure 1:**
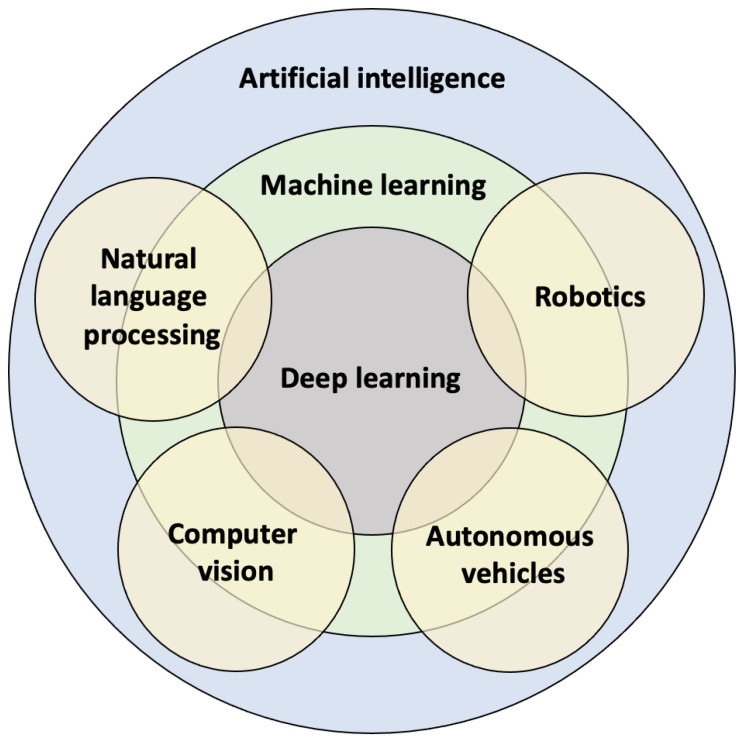
Artificial intelligence and its subfields.

Technological advances are changing many aspects of our society and industries, including healthcare. Innovative digital technologies such as computer-aided design/manufacturing, rapid prototyping, augmented/virtual reality, and “omics” analysis have been increasingly used in several medical and dental disciplines for diagnostic and therapeutic purposes [1]. Artificial intelligence (AI) is one of the most innovative and disruptive technologies that has the potential to revolutionize current clinical practice and research. The concept of AI was coined in the 1950s, referring to the idea of building machines that can imitate human behavior to perform complex tasks [2]. Since the 1950s, there were two “AI winters” in the 1970s and late 1980s, which occurred mainly due to insufficient computational power and resources, leading to a huge gap between the expectations and the actual outcomes of AI models of the time (i.e., knowledge-based expert systems) [2,3]. In the late 2000s, the advent of advanced graphics processing units reignited the enthusiasm for the development of various AI technologies and applications, such as intelligent robotics, autonomous vehicles, machine learning, computer vision, and natural language processing (Figure 1) [4].

Machine learning is a subfield of AI that enables algorithms to learn the intrinsic statistical patterns in a set of data without being explicitly programmed and then to make predictions on unseen data [2]. Due to the characteristics of data (e.g., clinical, biological, and radiographic data) and the major problems left unsolved in medicine and dentistry (e.g., early diagnosis, accurate prediction, and efficient treatment of specific diseases), machine learning and its subset deep learning are the most widely employed techniques in these fields [3]. Deep learning, a subset of machine learning, specifically uses multi-layered artificial neural networks to learn representations of data with multiple levels of abstraction [5]. Deep learning algorithms are trained to automatically extract hierarchical features in complex data and optimize the weighted parameters, leading to a more efficient learning process and minimized prediction errors. Within deep learning, the convolutional neural network (CNN) is a class of artificial neural networks that has been frequently used for image-related tasks, such as automated detection, segmentation, and classification of complex patterns in two-dimensional (2D) and three-dimensional (3D) images [3].

Radiomics is an emerging translational field in quantitative imaging, related to machine learning. It is capable of quantifying the textural information of specific regions of interest in digital diagnostic images through mathematic extraction of signal intensity distribution and pixel/voxel interrelationships that cannot be perceived by the human eye [6]. Based on high-throughput analysis of quantitative imaging features for the characterization of tissues, radiomics applications, similar to other omics clusters (e.g., genomics, proteomics, and metabolomics), have the potential to promote personalized diagnosis and management of interested diseases or conditions [5]. Currently, a wide range of radiomics models have been developed in many medical fields to assist clinicians in the screening, diagnosis, risk stratification, treatment response monitoring, and outcome prediction of malignancies, such as nasopharyngeal, lung, and breast cancer [6,7,8]. In general, AI and radiomics are interconnected and mutually reinforcing. AI, particularly machine learning, can serve as a powerful data modeling tool to analyze a massive number of radiomic features and parameters, while interpretation of radiomic features may provide information to better understand the decision-making process of a trained AI model.

Radiographic examination is an integral component in the diagnosis and management of most dento-maxillofacial diseases, and so a great amount of digital radiographic images are readily available in the archiving systems and associated databases of many hospitals and clinics. Hence, the majority of AI models in dentistry have been developed based on radiographic images to assist dentists in the diagnosis (e.g., detection of a certain pathology), treatment planning (e.g., segmentation of anatomical structures and pathologies), and prediction (e.g., classification of individuals with a higher possibility of good/poor treatment outcome) of various dental and maxillofacial diseases [3]. Computed tomography (CT) and cone-beam computed tomography (CBCT) are the most common 3D imaging modalities used in many dental specialties, especially in oral and maxillofacial surgery. Compared with conventional 2D dental radiography (i.e., periapical, bitewing, panoramic, and cephalometric radiography), CT/CBCT allows for the visualization of anatomical structures and pathologies in 3D, thus capable of providing not only higher diagnostic accuracy but also more detailed information regarding the true morphology, volume, and location of the lesions. As CBCT has gained increasing popularity in daily dental practice, this imaging modality is considered as an ideal data source for developing clinically practicable AI tools to improve the accuracy and efficiency of the diagnosis and treatment of maxillofacial diseases [9]. According to a recent systematic review, the number of AI models developed on CBCT images for dento-maxillofacial applications has increased dramatically over the years since the mid-2010s, particularly using deep learning [10]. Therefore, this narrative review aimed to provide an up-to-date overview of the current applications of deep learning and radiomics on CT and CBCT for the diagnosis and management of maxillofacial diseases.

## 2. Deep Learning and Radiomics on CT/CBCT for the Diagnosis and Management of Maxillofacial Diseases

Maxillofacial diseases include both odontogenic and nonodontogenic diseases in the jaws and related structures including salivary glands, temporomandibular joints (TMJs), and facial muscles. Due to their anatomical complexity and proximity to critical vascular and neural structures, 3D imaging (such as CT/CBCT) is often required in the diagnostic and treatment planning processes, serving as one of the essential components of digital workflows for patient management. Thus, a wide range of deep learning and radiomics applications have been developed based on CT/CBCT images for diagnosis, treatment planning, and prediction of various maxillofacial diseases (Table 1 and Table 2).

### 2.1. Jaw Cysts and Tumors

Incidental findings of lesions in the jaws are often observed on routine dental radiographic examination. These lesions are usually cystic in nature and thus challenging for general practitioners to differentiate between cysts and tumors on radiographic images. Motivated by the need for more timely and accurate diagnosis of maxillofacial cysts and tumors, several studies have developed AI, especially deep learning, models on 2D panoramic radiographs for automatic diagnosis of various cysts and tumors of the jaws [11,12,13,14,15]. Most of them focused on the lesions including ameloblastoma, odontogenic keratocysts (OKCs), dentigerous cysts, radicular cysts, and bone cysts, and the proposed models obtained high diagnostic accuracy on par with oral–maxillofacial surgeons.

Differentiating various types of jaw cysts and tumors on CBCT using deep learning has been proposed by several groups [16,17,18]. Lee et al. developed CNN models for automatic detection, segmentation, and classification of OKCs, dentigerous and periapical cysts on panoramic and CBCT images [16]. Not surprisingly, the model trained on CBCT images outperformed the one on panoramic radiographs, which may result from the advantages of CBCT in depicting the lesion morphology in 3D with more quantitative features in each voxel of the lesion region. Bispo et al. [17] and Chai et al. [18] developed CNN models to automatically classify between ameloblastoma and OKCs on CT images, respectively. Chai et al. compared the model’s performance with seven senior and thirty junior oral–maxillofacial surgeons. The model outperformed both senior and junior oral-maxillofacial surgeons with an accuracy of 85% in a short execution time of nearly 3 milliseconds per scan. These deep learning models have the potential to assist general dental practitioners in identifying different types of jaw cysts and tumors on CBCT images during daily dental practice, which would facilitate timely referral to oral–maxillofacial specialists and thus allow for the earliest possible treatment.

### 2.2. Lymph Node Metastasis

Oral squamous cell carcinoma (OSCC) is the sixth most frequent malignancy globally and associated with a high rate of invasiveness and lymph node metastasis [19]. Cervical lymph node metastasis has been reported as one of the major prognostic factors in OSCC patients that is directly correlated with a reduced overall survival rate. Patients with OSCC routinely have prophylactic neck dissection, as occult lymph node metastasis is not uncommon in clinical practice [20]. However, the neck dissection may result in severe functional and sensory complications (such as accessory or facial nerve paralysis and stiffness of the shoulder and neck), which considerably influence the patients’ quality of life [20]. Therefore, the controversy in the prophylactic neck dissection for patients with OSCC drives the search in non-invasive approaches with high accuracy in identifying lymph node metastasis. Contrast-enhanced CT is one of the most common imaging modalities used for the diagnosis of lymph node metastasis in OSCC patients. Size, necrosis, and extranodal extension are the main features for identifying cervical lymph node metastasis. However, complete detection of all metastatic lymph nodes using CT images based only on the perceivable nodal features is still challenging [21]. AI seems to be able to promote the development of a non-invasive approach for accurate identification of the nature of cervical lymph nodes in patients with OSCC.

Ariji et al. first developed a CNN model to differentiate histopathologically proven metastatic cervical lymph nodes from the negative ones on cropped contrast-enhanced CT images of OSCC patients [22]. The model achieved favorable accuracy with an AUC (area under the curve) of 0.80, which is similar to the level of two experienced radiologists (AUC of 0.83). Subsequently, Ariji et al. further proposed a CNN model to differentiate between metastatic lymph nodes with and without extranodal extension on cropped contrast-enhanced CT images of OSCC patients [23]. The model achieved high accuracy with an AUC of 0.82 and outperformed four experienced radiologists (AUCs of 0.52–0.63). However, these models could only make decisions on the cropped images of individual lymph nodes so that manual identification and cropping of all cervical lymph nodes on multiple CT image slices were required. Manual identification and cropping works are time-consuming, which would probably limit the implementation of these models for routine clinical use. In order to improve their clinical applicability, Ariji et al. developed CNN models for automated detection and segmentation of metastatic and non-metastatic cervical lymph nodes on contrast-enhanced CT images [24,25]. The latest model (AUC of 0.95) outperformed two experienced radiologists (AUC of 0.90) in detecting metastatic cervical lymph nodes while its segmentation accuracy should be further improved.

The potential of radiomics in the screening, diagnosis, and prediction of oral, head and neck cancer has been increasingly exploited [5]. Few studies investigated whether radiomic features extracted from CT/CBCT images could be used for predictive analysis of lymph node metastasis in patients with oral, head and neck cancer (Table 2). Kubo et al. developed predictive models based on CT-derived radiomic features using various machine learning classifiers for occult cervical lymph node metastasis in patients with tongue cancer [26]. The model trained with support vector machine (SVM) obtained the highest accuracy in predicting regional lymph node metastasis with an AUC of 0.98. Zhong et al. developed predictive models based on CT-derived radiomic features and associated clinical parameters for occult cervical lymph node metastasis in patients with tongue cancer [27]. The model on radiomic features and clinical lymph node status achieved higher prediction accuracy (AUC of 0.94) than the one only on radiomic features (AUC of 0.92). Morgan et al. developed several models based on CT- and CBCT-derived radiomic features and/or several clinical parameters using an explainable boosting machine for predicting local failure in head and neck cancer [28]. The model trained on both radiomic features and clinical parameters achieved the highest predictive performance with an AUC of 0.87 for primary cancer and 0.91 for nodal structures. The use of these models may enable a more personalized management of patients with oral, head and neck cancer.

### 2.3. Salivary Gland Diseases

Salivary gland diseases are a group of inflammatory, infectious, and neoplastic conditions, mainly occurring in the parotid glands, followed by minor salivary glands, submandibular glands, and sublingual glands. Diagnosis of salivary gland diseases is a great challenge in dentistry and oto-rhino-laryngology, as it heavily relies on the practitioners’ experiences and diagnostic imaging. Deep learning models for the diagnosis of salivary gland diseases have been developed largely on magnetic resonance imaging (MRI) scans [29] because of its advantages over other imaging modalities in depicting soft tissues. Nevertheless, MRI is not widely available in healthcare settings, and thus, CT remains an important role in identifying and describing the extent of neoplasms. Applying deep learning to CT images may expand its scope in the diagnosis of salivary gland diseases. Kise et al. developed a CNN model on CT images to automatically detect the fatty degeneration of the salivary gland that is a key characteristic finding for the diagnosis of Sjogren’s syndrome [30]. The model performed similarly to three experienced radiologists and outperformed three inexperienced radiologists. Additionally, deep learning models on CT achieved promising performance in differentiating various types of salivary gland tumors. Yuan et al. developed a CNN model to classify between pleomorphic adenoma and malignant parotid gland tumors on CT images [31]. The model achieved high classification accuracy of 90%. Zhang et al. trained several CNN models for classification between benign and malignant parotid gland tumors on CT images [32]. The customized CNN model achieved the highest classification accuracy of up to 98% as compared with the models built based on the pre-trained CNNs, including VGG16, InceptionV3, ResNet, and DenseNet.

The intrinsic radiomic features of malignant parotid gland tumors on CT images may be extracted to assist in differentiating between benign and malignant salivary gland tumors (Table 2). Zhang et al. developed a multivariable logistic regression model based on CT-derived radiomic features to classify between low- and high-grade mucoepidermoid carcinoma of the salivary glands [33]. The model showed that high-grade mucoepidermoid carcinomas may be associated with low energy, high correlation texture, and high surface irregularity. Xu et al. developed predictive models based on individual or combined CT-derived radiomic features and radiological variables (i.e., the location and metastases of lymph nodes) to differentiate between benign and malignant parotid gland tumors [34]. The model trained using SVM on CT-derived radiomic features and the location and metastases of lymph nodes achieved the highest performance than the models on individual radiomic features or radiological variables. Liu et al. developed predictive models based on either MRI or CT-derived radiomic features for differentiating between pleomorphic adenoma and Warthin tumors of the parotid glands, respectively [35]. The model on MRI-derived radiomic features (AUC of 0.91) performed slightly higher than but not significantly different from the model on CT-derived radiomic features (AUC of 0.88). These models may serve as auxiliary tools to assist clinicians in identifying patients with malignant salivary gland tumors.

### 2.4. Temporomandibular Joint Disorders

TMJ disorders are one of the most common orofacial dysfunctions that frequently cause joint clicking sounds, limited mouth opening, pain, and headache [36]. Degenerative bony changes is one of the main causes associated with TMJ disorders, which may start with the flattening or sclerosis of the mandibular condyle head, followed by the erosion of its cortical surface, and eventually osteoarthritis [37]. Accurate diagnosis of TMJ disorders is difficult for general dental practitioners and requires adequate clinical experience to avoid patients undergoing unnecessary examinations and invasive treatment. CBCT imaging is commonly used for assessing the morphology of TMJs. However, a low consistency among clinicians was found in the subjective interpretation of morphological changes in the TMJs [37], indicating that a quantitative diagnostic tool for TMJ disorders would be of great clinical value. Le et al. developed a CNN model for automated segmentation of the mandibular ramus and condyle on CBCT images [38]. Kim et al. developed a CNN model to automatically segment and measure the cortical thickness of the mandibular condyle head on CBCT images [39]. The model achieved favorable performance with a short execution time of nearly 10 s, which may contribute to automated quantitative analysis of the changes in bony structures of TMJs. de Dumast et al. developed a deep learning model on CBCT images to classify the morphological variations of TMJ osteoarthritis into five categories [40]. The model achieved high classification agreement of 91% with two human experts, demonstrating its potential to assist clinicians in the diagnosis of TMJ osteoarthritis. Bianchi et al. developed diagnostic models based on radiomic, biomolecular, clinical, and demographic features using different machine learning algorithms for TMJ osteoarthritis [41]. The model trained using the combination of XGBoost (Extreme Gradient Boosting) and LightGBM (Light Gradient Boosting Machine) algorithms achieved the highest accuracy with an AUC of 0.82. With the aid of this diagnostic model, screening individuals with early TMJ osteoarthritis seems possible.

### 2.5. Maxillary Sinus Pathologies

The maxillary sinus is the largest paranasal sinus and is frequently involved in various dento-maxillofacial surgical procedures, such as apical surgeries of the maxillary posterior teeth and sinus augmentation for dental implant placement [42,43]. Accurate diagnosis and classification of maxillary sinus pathologies prior to surgical procedures involving the sinus region is one of the key factors to ensure a predictable treatment outcome [44,45]. However, general practitioners may be less confident in diagnosing maxillary sinus pathologies on radiographic images due to their unfamiliarity with the anatomical structures and pathologies of the sinus. Implementation of dento-maxillofacial surgical procedures in the maxillary sinus with pathological changes are very likely to increase the risk of ostiomeatal dysfunction and sinus infection [46]. Some deep learning models on 2D radiographic images achieved favorable performance in detecting maxillary sinus pathologies [3]. One of the main limitations of the models on 2D images is their inculpability of providing information regarding the true morphology, volume, and location of the detected lesions, which are important in the planning of an appropriate treatment strategy for surgical procedures in the sinus region. Currently, CNN models were developed for automated segmentation of the maxillary sinus [47,48] and the morphological changes of the sinus mucosa [49,50] on CT/CBCT images. Jung et al. developed a CNN model on CBCT images to segment maxillary sinus pathologies including the mucosal thickening and mucous retention cysts of the sinus [49]. The model obtained favorable segmentation performance on internal data while its performance was inferior on external images. The scans including the maxillary sinus are frequently taken with a large field of view (FOV) and thus are associated with higher radiation exposure to the patients [51]. The use of a low-dose imaging protocol has been strongly recommended for large FOV scans of the sinus [52]. Hung et al. reported that their 3D CNN model achieved high performance on both standard-dose (AUCs of 0.89–0.93) and low-dose (AUCs of 0.84–0.89) CBCT images in automatic detection, segmentation, and measurement of the mucosal thickening and mucous retention cysts of the sinus [50]. No significant differences were found in the volume of the sinus, the lesions, and their ratio between automated and manual measurements. This CNN model has the potential to assist clinicians in identifying maxillary sinus lesions, evaluating the extent of sinus opacification, and planning surgical procedures in the sinus region.

### 2.6. Mandibular Fractures

The mandible is the only moveable and the most commonly fractured bone of the face in trauma cases. A CNN model was developed to automatically detect mandibular fractures on CT images [53]. The models first generated a synthesized panoramic image from the original CT scan. The mandibular region in the synthesized panoramic image was subsequently straightened and divided into image patches of nine subregions, including symphysis, left/right parasymphysis, left/right mandibular body, left/right mandibular angle, and left/right condylar process. Eventually, the model determined the presence or absence of a fracture on the image patches of specific mandibular subregions. This model achieved high fracture detection accuracy with AUC values of 0.93–0.98 across the nine mandibular subregions, which may be particularly useful for detecting occult condylar fractures.

### 2.7. Dentofacial Deformities and Malocclusion

Dentofacial deformities and malocclusion are characterized by abnormalities of the dentition, jaws, and face that affect not only the oral function and appearance of patients but also their physical, social, and psychological well-being. Orthodontic and orthognathic treatment is commonly performed to correct these abnormalities. Conventional treatment planning of orthognathic surgery consists of a series of steps based on clinical examination, cephalometric analysis, dental casts, facebow, and articulators. Compared with the conventional approach, a digital workflow improves the accuracy and efficiency of orthognathic surgery without the need of a facebow record and model surgery [54].

**Figure 2:**
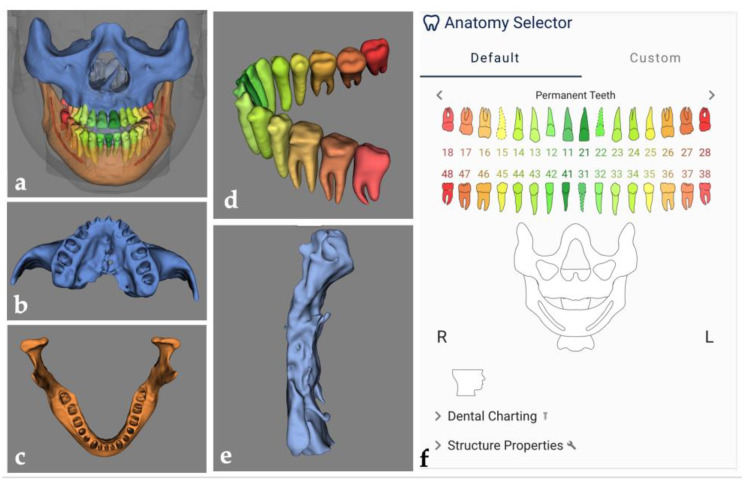
Example of automated segmentation of dento-maxillofacial anatomical structures on CBCT images using a commercially available AI software platform, Relu (Leuven, Belgium; available at https://relu.eu (accessed on 5 December 2022)). The overview of the segmented anatomical structures (**a**), including the maxilla (**b**), mandible (**c**), teeth with orthodontic brackets (**d**), and pharyngeal airway (**e**), and automated labeling of teeth (**f**).

The digital workflows in dentistry frequently require a 3D virtual augmented patient model that is created based on multimodal image data (such as CBCT and optical intra-oral, facial, and/or model scans) to serve as the foundation for subsequent treatment planning and guided surgery for many surgical procedures including orthognathic surgery. Segmentation of anatomical structures and multimodal image registration are the essential steps in the process of building a virtual patient model. They can be performed manually through visual inspection or semi-automatically by using the fiducial marker registration method or surface-based matching algorithm [55]. The manual approach is time-consuming, and errors by visual inspection are unavoidable. While the semi-automatic approach can improve the efficiency of these steps, manual correction is still necessary prior to further planning steps, such as the design of surgical splints for orthognathic corrections. Deep learning models for automatically segmenting anatomical structures on CBCT images or merging the contour of the interested region from different image datasets may be a solution to improving the accuracy and efficiency of image segmentation and registration. Commercially available AI platforms, such as CranioCatch (Eskişehir, Turkey), Denti.AI (Toronto, ON, Canada), Diagnocat (Tel Aviv, Israel), Promaton (Amsterdam, The Netherlands), and Relu (Leuven, Belgium), and several CNN models have been proposed for automated segmentation of dento-maxillofacial anatomical structures (including teeth, jaws, maxillary sinus, pharyngeal airway, mandibular canal, palatine, zygomatic, nasal, and lacrimal bones) on CBCT images (Figure 2) [56,57,58,59,60,61,62,63]. Some of them performed similarly to experienced radiologists and outperformed other semi-automatic software, such as Mimics^®^ (version 23.0, Materialise N.V., Leuven, Belgium). Automated multimodal image registration to merge CBCT and optical intra-oral/model scans using deep learning has been proposed by some groups [64,65]. The model by Jang et al. showed high accuracy with a mean registration error of 0.5 mm, which is less than that of the manual registration approach (1.7 mm) [64]. The model by Chung et al. completed the image registration procedure in a short period of nearly 20 s with registration errors less than that of the conventional three-point registration method [65]. The model’s performance was not affected by the presence of metal artifacts on CBCT images, which may greatly improve image registration accuracy in patients with multiple metallic dental restorations.

In addition, deep learning was applied to determine whether an individual needs orthognathic surgery and to predict the surgical outcomes. Kim et al. developed several CNN models on CBCT images to automatically classify individuals into Class I, II, and III skeletal malocclusion according to several parameters including the anteroposterior relationship of the maxillary and mandibular first molars and the alignment of teeth with reference to the line of occlusion [66]. The performance of the developed models was up to an accuracy of 93.8%. The model may facilitate orthognathic evaluation by identifying individuals in need of surgical correction of skeletal malocclusion. Few CNN models were developed on CT/CBCT images to predict the changes in the skeletal and soft-tissue profile after orthognathic surgery [67,68,69]. The model by ter Horst et al. for soft-tissue profile prediction performed similarly to a soft tissue prediction model (mass tensor model) that is widely used for maxillofacial surgical planning [67]. These predictive models may assist surgeons in orthognathic surgical planning to improve postoperative facial symmetry.

**Table 1:** Performance of deep learning models on CT/CBCT images for the diagnosis and treatment planning of maxillofacial diseases.

Author (Year)	Application	Imaging Modality	Model/Platform	Training and Validation Dataset	Test Dataset/Cross-validation	Execution Time	Performance		Major Findings
							Deep Learning	Manual/Semi-automatic Method	
Jaw cysts and tumors									
Lee et al. (2020) [16]	Detection, segmentation, and classification of OKCs, dentigerous and periapical cysts	Panoramic and CBCT images	CNN (Inception v3)	912 panoramic and 789 CBCT images	228 panoramic and 197 CBCT images	NA	Panoramic/CBCT AUC = 0.85/0.91 SEN = 88%/96% SPE = 77%/77%	NA	The model on CBCT images obtained higher diagnostic performance than the one on panoramic images.
Bispo et al. (2021) [17]	Differential diagnosis of ameloblastoma and OKCs	CT	CNN (Inception v3)	2500 images augmented based on 350 slices from 40 scans of patients with ameloblastoma or OKCs	2-fold CV with 5 iterations	NA	ACC = 90–92%	NA	The model obtained higher accuracy in identifying OKCs than ameloblastoma.
Chai et al. (2022) [18]	Classification of ameloblastoma and OKCs	CBCT	CNN (Inception v3)	272 scans of patients with ameloblastoma or OKCs	78 scans of patients with ameloblastoma or OKCs	Model/Senior/Junior OMF surgeons 36/1471/1113 s (78 scans)	ACC = 85% SEN = 87% SPE = 82% F1 = 85%	7 senior/30 junior OMF surgeons ACC = 66%/59% SEN = 60%/64% SPE = 71%/53% F1 = 64%/61%	The model outperformed both senior and junior OMF surgeons.
Lymph node metastasis									
Ariji et al. (2019) [22]	Differentiation of metastatic cervical lymph nodes from negative lymph nodes in OSCC patients	Contrast-enhanced CT	CNN (AlexNet)	441 cropped images including 127 metastatic and 314 non-metastatic lymph nodes from 45 OSCC patients	5-fold CV	NA	AUC = 0.80 ACC = 78% SEN = 75% SPE = 81% PPV = 80% NPV = 77%	2 radiologists AUC = 0.83 ACC = 83% SEN = 78% SPE = 89% PPV = 87% NPV = 80%	The model performed similarly to the radiologists.
Ariji et al. (2020) [23]	Differentiation between metastatic lymph nodes with and without extranodal extension in OSCC patients	Contrast-enhanced CT	CNN (AlexNet)	80% of 703 cropped images including metastatic lymph nodes with or without extranodal extension from 51 OSCC patients	20% of 703 cropped images	11 s	AUC = 0.82 ACC = 84% SEN = 67% SPE = 90% PPV = 69% NPV = 89%	4 Radiologists AUC = 0.52–63 ACC = 51–63% SEN = 42–55% SPE = 57–71% PPV = 52–66% NPV = 51–61%	The model outperformed 4 radiologists in identifying metastatic lymph nodes with extranodal extension.
Ariji et al. (2021) [24]	Detection of cervical lymph nodes in OSCC patients	Contrast-enhanced CT	CNN (DetectNet)	320 image slices including 134 metastatic and 448 non-metastatic lymph nodes from 56 OSCC patients	45 image slices including 25 metastatic and 69 non-metastatic lymph nodes from 56 OSCC patients	8 s	SEN = 73% PPV = 96% F1 = 83% False positive rates per images = 4%	NA	The model has the potential to automatically detect cervical lymph nodes.
Ariji et al. (2022) [25]	Detection and segmentation of metastatic cervical lymph nodes in OSCC patients	Contrast-enhanced CT	CNN (U-Net)	911 image slices including 134 metastatic and 446 non-metastatic lymph nodes from 59 OSCC patients	72 image slices of 24 metastatic and 68 non-metastatic lymph nodes from 59 OSCC patients	7 s	Detection AUC = 0.95 ACC = 96% SEN = 98% SPE = 95% Segmentation SEN = 74% PPV = 94% F1 = 83%	2 radiologists Detection AUC = 0.90 ACC = 89% SEN = 94% SPE = 86%	The model outperformed 2 radiologists in detecting metastatic cervical lymph nodes while its segmentation accuracy should be improved.
Salivary gland diseases									
Kise et al. (2019) [30]	Diagnosis of Sjögren’s syndrome	CT	CNN (AlexNet)	400 image slices from 20 scans of patients with Sjögren’s syndrome and 20 scans of individuals without parotid gland abnormalities	100 image slices from 5 scans of patients with Sjögren’s syndrome and 5 scans of individuals without parotid gland abnormalities	NA	ACC = 0.96 SEN = 100% SPE = 92%	3 experienced/3 inexperienced OMF radiologists ACC = 98%/84% SEN = 99%/78% SPE = 97%/89%	The model performed similarly to experienced radiologists and outperformed inexperienced radiologists.
Zhang et al. (2021) [32]	Classification between benign and malignant parotid gland tumors	CT	CNNs (Improved CNN, VGG16, InceptionV3, ResNet, and DenseNet)	720 image slices (group 1) and 1050 image slices (group 2)	180 image slices (group 1) and 270 image slices (group 2)	<1 min	Improved CNN on Group 1/2 ACC = 98%/78% SEN = 97%/77% SPE = 99%/79% PPV = 99%/79% F1 = 98%/78%	NA	The improved CNN model achieved the highest classification accuracy than other pre-trained CNN models.
Yuan et al. (2022) [31]	Classification between pleomorphic adenoma and malignant parotid gland tumors	CT	CNN (ResNet50)	121 scans	30 scans	NA	ACC = 90%	NA	The model achieved high accuracy in identifying malignant parotid gland tumors.
Temporomandibular disorders									
de Dumast et al. (2018) [40]	Classification of morphological variation in TMJ osteoarthritis	CBCT	Deep neural network	Scans of 259 condyles from 154/105 individuals with/without TMJ osteoarthritis	Scans of 34 condyles from 17/17 individuals with/without TMJ osteoarthritis.	NA	Agreement with two experts = 91%	NA	The model has the potential to assist clinicians in the diagnosis of TMJ osteoarthritis.
Kim et al. (2021) [39]	Segmentation and measurement of the cortical thickness of mandibular condyle head	CBCT	CNN (U-Net)	11,776 image slices from 23 scans of individuals without pathological bony changes on the condyle head	1024 image slices from 2 scans of individuals without pathological bony changes on the condyle head	10–15 s	Marrow bone IoU = 0.87 HD = 0.93 mm Cortical bone IoU = 0.73 HD = 1.25 mm	NA	The model may contribute to automated quantitative analysis of the changes in bony structures of TMJ.
Le et al. (2021) [38]	Segmentation of mandibular ramus and condyle	CBCT	CNN (U-Net)	90 scans of individuals with/without osteoarthritis, obtained from multiple centers	19 scans of individuals with/without osteoarthritis, obtained from multiple centers	NA	AUC = 0.95 ACC = 100% SEN = 93% SPE = 100% F1 = 92%	NA	The model may facilitate treatment planning of TMJ degeneration.
Maxillary sinus									
Xu et al. (2020) [47]	Segmentation of the maxillary sinus	CT	CNN (V-Net)	35 scans	26 scans	<1 min	DSC = 0.94 IoU = 0.90 Precision = 94%	NA	The model achieved high segmentation accuracy.
Deng et al. (2020) [48]	Segmentation of the maxillary sinus	CT	CNN (BE-FNet)	50 scans	5-fold CV	0.5 s	DSC = 0.95 VOE = 10.2% ASD = 2.9 mm	NA	The model achieved high segmentation accuracy.
Jung et al. (2021) [49]	Segmentation of maxillary sinus lesions	CBCT	CNN (3D nnU-Net)	83 scans obtained from Korea University Anam Hospital	20 scans obtained from Korea University Anam Hospital and 20 scans from Korea University Ansan Hospital	NA	Anam Hospital DSC = 0.76 Ansan Hospital DSC = 0.54	NA	A lower segmentation accuracy of the model was found on external images.
Hung et al. (2022) [50]	Detection, segmentation, and measurement of the morphological changes of the sinus mucosa	CBCT	CNN (V-Net and SVR)	347 low-dose scans of individuals with or without morphological changes of the maxillary sinus mucosa	77 low-dose and 21 standard-dose scans of individuals with or without morphological changes of the maxillary sinus mucosa	NA	Low-dose scans AUC = 0.84–0.89 SEN = 79–81% SPE = 71–89% Standard-dose scans AUC = 0.89–0.93 SEN = 79–93% SPE = 89–93%	NA	The model performed similarly on both standard- and low-dose scans.
Fractures									
Wang et al. (2022) [53]	Detection and classification of mandibular fractures	CT	CNNs (U-Net and ResNet)	278 scans	408 scans	NA	AUC = 0.93–0.98 ACC = 94–98% SEN = 91–97% SPE = 91–99%	NA	The model may assist clinicians in timely and accurate detection of mandibular fractures.
Dentofacial deformities and malocclusion									
Kim et al. (2020) [66]	Classification of skeletal malocclusion	CBCT	Multi-channel CNNs	173 scans of individuals with Class I, II, or III malocclusion	45 scans of individuals with Class I, II, or III malocclusion	NA	ACC = 93–94% SEN = 95% PPV = 93–94% F1 = 94–95%	NA	The model may facilitate orthodontic and orthognathic evaluation to determine whether the patient needs surgical correction.
Ma et al. (2022) [68]	Prediction of skeletal changes after orthognathic surgery	CT	CNN	50 pairs of preoperative and postoperative full skull scans	6 pairs of preoperative and postoperative full skull scans	43 s	Mean landmark localization deviation = 5.4 mm 74% of the predicted postoperative skull models was consistent with the ground truth	NA	The model may assist OMF surgeons in predicting postoperative skeletal changes for orthognathic surgical planning.
ter Horst et al. (2021) [67]	Prediction of virtual soft tissue profile after mandibular advancement surgery	3D photographs and CBCT	Autoencoder-inspired neural network	119 pairs of 3D photographs and CBCT scans of patients who underwent mandibular advancement surgery	14 pairs of 3D photographs and CBCT scans of patients who underwent mandibular advancement surgery	NA	Mean absolute error 1 mm (lower face) 1.1 mm (lower lip) 1.4 mm (chin)	MTM-based soft-tissue simulations Mean absolute error 1.5 mm (lower face) 1.7 mm (lower lip) 2 mm (chin)	The model performed similarly to the MTM-based soft-tissue simulations, indicating that it may be useful for soft tissue profile prediction in orthognathic surgery.
Lin et al. (2021) [69]	Assessment of facial symmetry before and after orthognathic surgery	CBCT	CNNs (VGG16, VGG19, ResNet50, and Xception)	71 scans	59 scans	NA	ACC 80% (VGG16) 86% (VGG19) 83% (ResNet50) 90% (Xception)	NA	The model trained with Xception achieved highest accuracy for facial symmetry assessment.
Image registration									
Chung et al. (2020) [65]	Registration between CBCT and optical dental model scans	CBCT and optical dental model scans	Deep pose regression neural networks and optimal cluster-based matching	150 pairs of CBCT and optical maxillary model scans and 150 pairs of CBCT and mandibular model scans	3-fold CV	17.6 s	Mean distance errors 5.1 mm (surface) 1.8 mm (landmarks)	Conventional three-point registration Mean distance errors 9.6 mm (surface) 2.7 mm (landmarks)	The model is applicable to full-arch scanned models and can avoid metal artifacts during the matching procedures.
Jang et al. (2021) [64]	Registration between CBCT and intraoral scans	CBCT and intraoral scans	CNN	71 maxillary or mandibular intraoral scans and the corresponding 49 CBCT scans	22 pairs of CBCT and intraoral scans	NA	Mean distance errors 0.5 mm (surface) 0.2 mm (landmarks)	Manual registration Mean distance errors 1.7 mm (surface) 0.7 mm (landmarks)	The model outperformed the manual registration method.
Segmentation of maxillofacial structures									
Lo Giudice et al. (2021) [56]	Segmentation of the mandible	CBCT	CNN	20 scans	20 scans	50 s	DSC = 0.97 Matching percentage = 89%	NA	The model may be useful in the planning of maxillofacial surgical procedures.
Xu et al. (2021) [63]	Segmentation of mandibles with/without tumor invasion	CT	CNN (3D V-Net)	160 scans of 80 consisting of 80 MTI scans and 80 Non-MTI scans	70 scans consisting of 35 MTI scans and 35 Non-MTI scans	7.4 s	Non-MTI segmentation DSC = 0.98 IoU = 0.96 ASD = 0.06 mm HD = 0.48 mm MTI segmentation DSC = 0.97 IoU = 0.94 ASD = 0.16 mm HD = 1.16mm	NA	The model obtained high accuracy in segmenting mandibles with and without tumor invasion.
Sin et al. (2021) [59]	Segmentation of pharyngeal airway	CBCT	CNN (U-Net)	260 scans	46 scans	NA	DSC = 0.92 IoU = 0.99	NA	The model can efficiently calculate the pharyngeal airway volume from CBCT images.
Orhan et al. (2022) [60]	Segmentation of the pharyngeal airway in OSA and non-OSA patients	CBCT	Diagnocat (a commercially available AI platform; https://diagnocat.com (accessed on 5 December 2022))	NA	200 scans of 100 OSA and 100 non-OSA patients, taken using 3 different CBCT scanners	NA	ICC between Diagnocat and radiologists = 0.97	NA	Diagnocat performed similarly to radiologists and can efficiently calculate the pharyngeal airway volume in OSA and non-OSA patients.
Preda et al. (2022) [57]	Segmentation of the maxillofacial complex, including palatine, maxillary, zygomatic, nasal, and lacrimal bones	CBCT	CNN (U-Net)	120 scans taken using two different scanners	24 scans taken using two different scanners	Model 39 s Manual 133 min	DSC = 0.93 IoU = 0.86 95% HD = 0.62 mm RMS 0.5 mm	Semi-automated segmentation using Mimics DSC = 0.69 IoU = 0.53 95% HD = 2.78 mm RMS 1.76 mm	The model may improve the efficiency of the digital workflows for patient-specific treatment planning of maxillofacial surgical procedures.
Ezhov et al. (2021) [58]	Segmentation of teeth and jaws, numbering of teeth, detection of caries, periapical lesions, and periodontitis	CBCT	Diagnocat (a commercially available AI platform; https://diagnocat.com (accessed on 5 December 2022))	1346 scans taken using 17 scanners	30 scans	With the aid of Diagnocat = 17.6 min Without the aid of Diagnocat = 18.7 min	Diagnocat SEN = 92% SPE = 99% 12 dentists with the aid of Diagnocat SEN = 85% SPE = 97%	4 OMF radiologists SEN = 93–94% SPE = 99–100% 12 dentists without the aid of Diagnocat SEN = 77% SPE = 96%	Diagnocat performed similarly to four radiologists and improved twelve dentists’ performance
Jaskari et al. (2020) [61]	Segmentation of the mandibular canal	CBCT	CNN	509 scans taken using two scanners	15 scans	NA	MCD = 0.56 mm ASSD = 0.45 mm DSC = 0.57 (left) and 0.58 (right) HD = 1.40 (left) and 1.38 (right)	NA	The model may help to locate the inferior alveolar nerve for surgical planning
Lim et al. (2021) [62]	Segmentation of the mandibular canal	CBCT	CNN (3D nnU-Net)	83 scans from Korea University Anam Hospital	15, 20, and 20 scans from Korea University Anam Hospital (1), Korea University Ansan Hospital (2), and Korea University Guro Hospital (3)	Model 86 s Manual 125 s	Internal testing DSC = 0.58 (1) External testing DSC = 0.55 (2) DSC = 0.43 (3)	NA	The model may help to locate the inferior alveolar nerve for surgical planning

Abbreviations: 3D, three-dimensional; ACC, accuracy; ASSD, average symmetric surface distance; AUC, area under the ROC curve; CBCT/CT, cone-beam computed tomography; CMS, contour matching score; CNN, convolutional neural network; CV, cross-validation; DSC, dice similarity coefficient; F1, F1-score; HD, hausdorff distance; IoU, intersection over union; JSC, jaccard similarity coefficient; k-NN, k-nearest neighbors; OKC, odontogenic keratocyst; LDA, linear discriminant analysis; LOOCV, leave-one-out cross-validation; MCD, mean curve distance; MTM, mass tensor model; MTI, mandible with tumor invasion; NA, not available; NN, neural network; NPV, negative predictive value; OMF, oral and maxillofacial; OSA, obstructive sleep apnea; OSCC, oral squamous cell carcinoma; PPV, positive predictive value (precision); RMS, root mean square; SDA, sparse discriminant analysis; SEN, sensitivity (recall); SPE, specificity; SSIM, structural similarity index measure; SVM, support vector machine; TMJ, temporomandibular joint; VOE, volumetric overlap error.

**Table 2:** Performance of radiomics models on CT/CBCT images for maxillofacial diseases.

Author (Year)	Application	Imaging Modality	Image Dataset	Region of Interest for Feature Extraction	Data for Model Building	Machine Learning Approach	Validation Method	Performance of the Best Model(s)	Major Findings
Zhong et al. (2021) [27]	Prediction of cervical lymph node metastasis in patients with tongue cancer	Contrast-enhanced CT	313 scans of patients with tongue cancer	Primary cancer	Radiomic features and clinical lymph node status	Artificial neural network	Hold-out validation (20%)	Model on radiomic features and clinical lymph node status AUC = 0.94 ACC = 84% SEN = 93% SPE = 77% Model on radiomic features AUC = 0.92 ACC = 86% SEN = 82% SPE = 89%	The model on radiomic features and clinical lymph node status achieved higher prediction accuracy than the one only on radiomic features.
Kubo et al. (2022) [26]	Prediction of occult cervical lymph node metastasis in patients with tongue cancer	Contrast-enhanced CT	161 scans of tongue cancer patients with or without occult cervical lymph node metastasis	Cervical lymph nodes	Radiomic features	kNN, SVM, CART, RF, AdaBoost with/without SMOTE	10-fold CV	Side level RF with SMOTE AUC = 0.92 ACC = 85% SEN = 82% PPV = 88% Region level SVM with SMOTE AUC = 0.98 ACC = 96% SEN = 95% PPV = 96%	The radiomics models may serve as useful tools to support clinical decision making in the management of patients with tongue cancer.
Morgan et al. (2021) [28]	Prediction of local failure in head and neck cancer	Contrast-enhanced CT and CBCT	Baseline CT scan, two CBCT scans at fractions 1 and 21 of radiotherapy from 90 head and neck SCC patients with or without local failure	All primary and nodal structures	Radiomic features and several clinical variables	Explainable boosting machine with 25 iterations	5-fold CV	Fused ensemble model (primary/nodal structures) AUC = 0.87/0.91 SEN = 78%/100% SPE = 91%/68%	The model on radiomic features and clinical variables achieved the highest accuracy in predicting local failure in head and neck cancer.
Xu et al. (2021) [34]	Differentiation between benign and malignant parotid gland tumors	CT	87 scans of patients with benign or malignant parotid gland tumor	Primary tumors	Radiomic features and radiological variables including the location and metastases of lymph nodes	SVM	Hold-out validation (38 scans)	The combined model AUC = 0.84 SEN = 82% SPE = 74% The model on radiomic features AUC = 0.77 SEN = 79% SPE = 89%	The combined model outperformed the models on individual radiomic features, lymph node location, or lymph node metastases.
Zhang et al. (2021) [33]	Differentiation between low- and high-grade mucoepidermoid carcinoma of the salivary glands	CT	53 scans of patients with low or high grade mucoepidermoid carcinoma	Primary cancer	Radiomic features	Logistic regression	NA	AUC = 0.80 ACC = 78% SEN = 89% PPV = 67%	High-grade mucoepidermoid carcinomas may be associated with a low energy, high correlation texture, and high surface irregularity.
Liu et al. (2021) [35]	Differentiation between pleomorphic adenoma and Warthin tumors of the parotid glands	CT and MRI	659 pairs of CT and MRI scans from patients with pleomorphic adenoma or Warthin tumors	Primary tumors	CT- and MRI-derived radiomic features	Logistic regression	NA	CT/MRI AUC = 0.88/0.91 ACC = 78%/84% SEN = 81%/85% SPE = 76%/83% PPV = 70%/77% NPV = 86%/89%	The model on MRI-derived radiomic features performed slightly higher than but not significantly differently from the model on CT-derived radiomic features.
Bianchi et al. (2020) [41]	Diagnosis of TMJ osteoarthritis	CBCT	92 scans of subjects with or without TMJ osteoarthritis	Internal condylar lateral region	20 radiomic and 25 biomolecular features, 5 clinical and 2 demographic variables	LR, RF, LightGBM, XGBoost with 10 iterations	5-fold CV	XGBoost + LightGBM AUC = 0.87 ACC = 82% SEN = 84% F1 = 82%	The model may be helpful for screening individuals with early TMJ osteoarthritis.

Abbreviations: ACC, accuracy; AUC, area under the ROC curve; CART, classification and regression tree; CBCT/CT, cone-beam computed tomography; CV, cross-validation; F1, F1-score; LASSO, least absolute shrinkage and selection operator; LightGBM, light gradient boosting machine; LR, logistic regression; mRMR, maximum relevance and minimum redundancy; kNN, k-nearest neighbor; ICC, intra-class correlation coefficient; MRI, magnetic resonance imaging; PPV, positive predictive value (precision); RF, random forest; RFE, recursive feature elimination; SCC, squamous cell carcinoma; SEN, sensitivity (recall); SMOTE, synthetic minority oversampling technique; SPE, specificity; SVM, support vector machine; TMJ, temporomandibular joint; U test, Mann–Whitney U test; XGBoost, extreme gradient boosting.

## 3. The Challenges and Prospects of Deep Learning and Radiomics on CT/CBCT for Maxillofacial Diseases

Based on current evidence, early diagnosis, accurate prognostic prediction, and efficient treatment planning are main focuses of deep learning and radiomics models developed on CT/CBCT for maxillofacial diseases (Table 1 and Table 2). Few studies reported that deep learning models on CBCT images performed better than those on 2D radiographic images [3,16]. These findings may result from more informative features on CBCT than on 2D images to be utilized for training the models. Most of the proposed deep learning models showed high performance, and some of them even outperformed human experts, especially when the ground truth was not based solely on visual inspection on radiographic images. Deep learning models capable of detecting diseases, particularly malignant lesions, at an early stage are expected to allow for the earliest possible diagnosis and treatment to prevent disease progression, which therefore will improve treatment outcome and prognosis. Apart from diagnostic applications, deep learning models were also developed to assist clinicians in many time-consuming tasks required in the treatment planning process for patients with maxillofacial diseases as mentioned above. Applications for automated multimodal image registration as well as localization, segmentation, and measurement of anatomical structures or pathologies on CT/CBCT images have the potential to improve the accuracy and efficiency of digital workflows for patient-specific treatment planning, which may enable a more precise and personalized approach for the management of maxillofacial diseases.

Despite the promising performance of deep learning models proposed, their generalizability has not been validated sufficiently. Most of them were trained using CT/CBCT images acquired at a certain time point from a single institution and were tested with the cross-validation or split sample validation method using images from the same institution, which is very likely to cause overfitting of the trained model. Some studies have reported that their models had inferior performance when tested on images from other institutions [70,71]. More validation studies that prospectively collect new datasets to test the performance of the developed models are needed. Ideally, the model’s performance should be evaluated on external image data, acquired with different scanners and imaging protocols, from multiple institutions to verify their true generalizability. If the model’s performance on external datasets is not favorable, the datasets from different centers should be included for cross-center training to avoid overfitting and improve the model’s generalizability. On the other hand, training data insufficiency is also one of the most common reasons that cause overfitting, resulting in the model’s learning statistical regularity specific to the training data. Some strategical learning approaches, such as federated learning and learning from the normal methods, may be the solution to overcome the insufficiency of training data [72]. Moreover, it has been raised that radiomic analysis is more robust than deep learning approach in the case of training with small data [73]. Incorporating radiomic features into deep learning models seems to be able to avoid overfitting [73].

**Figure 3:**
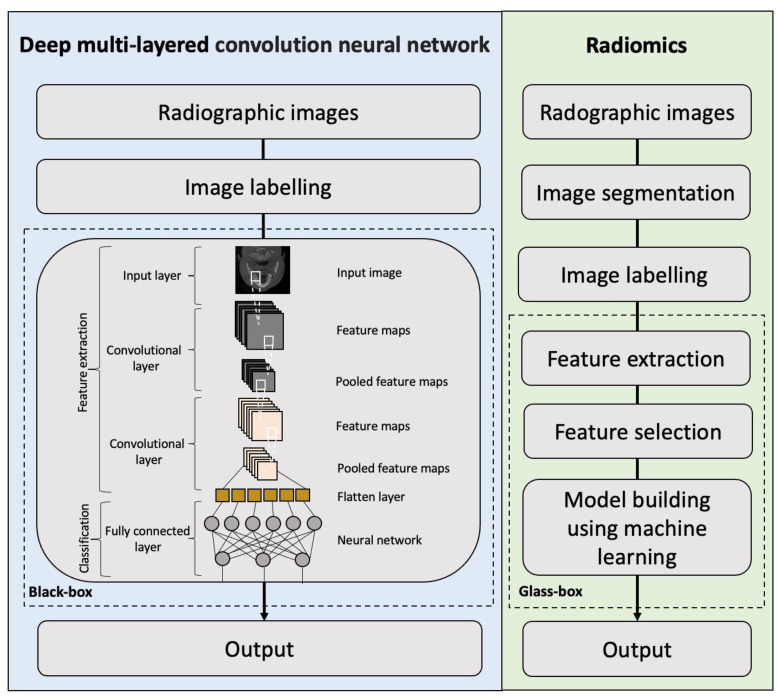
Flowchart demonstrating the main differences in the workflow between deep learning and radiomics in radiological studies.

Deep learning algorithms allow for automatic extraction and selection of imaging features on radiographic images in the neural network. As deep learning models automatically extract hierarchical features in complex data and optimize the weighted parameters from raw data, their decision-making process cannot be deduced, and thus, they are considered as “black-box” models (Figure 3). Compared with deep learning models, radiomics models have been seen as “glass-box” models because of better transparency [28]. The radiomics approach involves the extraction of quantitative imaging features from the segmented regions of interest on radiographic images, selection of reproducible and reliable features, and building a high-level statistical model with the selected features using machine learning methods for diagnostic and predictive purposes. Therefore, the contribution of each selected feature to the overall prediction can be deduced from radiomics models, which is one of the main advantages of radiomic analysis as compared to deep learning [28]. Thus far, radiomics studies on CT/CBCT were conducted mainly for differentiating between benign and malignant lesions as well as predicting cervical lymph node metastasis and local failure in patients with oral, head and neck cancer. There are still several challenges in current radiomics studies regarding the repeatability and reproducibility of radiomic features and the stability of feature selection [74,75,76]. The variations in the scanners, imaging protocols, and reconstruction algorithms may affect the repeatability and reproducibility of radiomic features [77]. Moreover, radiomics models built based on an unstable feature selection method may include many unstable features, resulting in a lack of reliability of the developed models and reduced accuracy on external data. The use of ensemble methods, including resampling, bagging, and boosting techniques, for radiomic feature selection has been highly recommended to improve the stability of radiomic feature selection [28,78]. Regardless, radiomic models also have some limitations when compared to deep learning methods, such as the requirement of segmentation, and its application is limited to classification of segmented lesions. These limitations may be overcome by integrating radiomics and deep learning to expand their clinical applications.

It remains unknown whether CT- and CBCT-derived radiomic features are interchangeable. Few studies have assessed the differences in radiomic feature values of head and neck cancer between CT and CBCT images of the same individuals [79,80]. It was reported that no significant differences were found in most of the extracted feature values between the paired CT and CBCT images, indicating that radiomic features from CT and CBCT may be interchangeable [79]. Notably, some image processing techniques, such as high-pass filtering, could affect the reproducibility of radiomic features [79]. On the contrary, some held the view that radiomic features from CBCT may not be directly transferable to those from CT due to the differences in their inherent image characteristics, such as the scatters, noise, and resolution [80]. These differences may contribute to larger variations in radiomic feature values calculated from specific regions of interest between the two imaging modalities. The analysis of delta radiomic features (i.e., the changes in radiomic feature values from serial scans) may be the solution to improve the reproducibility of radiomic features for the management of oral, head and neck cancer [80].

The reproducibility in the radiomic feature values calculated by different software packages (such as Pyradiomics, MaZda, LIFEx, MITK Phenotyping, and CERR radiomic extension) remains uncertain. Some found that the values of features in certain categories (e.g., second-order features) were not consistent across packages [81] while others reported high consistency [82]. Researchers should be aware of this issue when comparing results from studies using different radiomics software packages. The image biomarker standardization initiative (IBSI; https://ibsi.readthedocs.io/ (accessed on 5 December 2022)) is an independent international collaboration where experts in various areas of medical imaging from several institutions in eight countries work together to standardize the extraction of image biomarkers (i.e., radiomic features) from diagnostic imaging for the purpose of achieving greater harmonization of radiomics research [83]. Standardization of radiomic analysis is fundamental for the comparison and validation of findings from different studies and is crucial for a possible translation of radiomics into clinical practice.

At present, most of the deep learning and radiomics models for maxillofacial diseases were developed based solely on CT/CBCT image data. Enriching these models with diverse data from the individual level (such as demographic, behavioral, and social characteristics), setting level (such as geospatial, environmental, or provider-related data), and system level (such as health insurance, regulatory, and legislative data) may facilitate a deeper and more holistic understanding of individual health and disease and may therefore enable a more precise and personalized management of patients with maxillofacial diseases [84]. Most importantly, the true usefulness and cost-effectiveness of these deep learning and radiomics models in daily practice should be further assessed to gain the trust of patients, providers, and healthcare organizers. Further development of explainable AI systems that can provide an insight of how the predictions are made is the key to fostering trust in their clinical use [73].

## 4. Conclusions

A wide range of deep learning and radiomic models on CT/CBCT have been proposed for automatic diagnosis, segmentation, and classification of jaw cysts and tumors, cervical lymph node metastasis, salivary gland diseases, TMJ disorders, maxillary sinus pathologies, mandibular fractures, and dentomaxillofacial deformities. The models with performance on par with specialists have the potential to serve as clinically practicable tools to achieve the earliest possible diagnosis and treatment, leading to a more precise and personalized approach for the management of maxillofacial diseases.
